# TIP60-dependent acetylation of the SPZ1-TWIST complex promotes epithelial–mesenchymal transition and metastasis in liver cancer

**DOI:** 10.1038/s41388-018-0457-z

**Published:** 2018-08-28

**Authors:** Li-Ting Wang, Shen-Nien Wang, Shyh-Shin Chiou, Kwei-Yan Liu, Chee-Yin Chai, Cheng-Ming Chiang, Shau-Ku Huang, Kazunari K. Yokoyama, Shih-Hsien Hsu

**Affiliations:** 1 Graduate Institute of Medicine, Kaohsiung Medical University, Kaohsiung 807, Taiwan; 2 Department of Surgery, Kaohsiung Medical University Hospital, Kaohsiung 807, Taiwan; 3 Pingtung Hospital, Ministry of Health and Welfare, Pingtung 900, Taiwan; 4 Department of Pediatrics, Kaohsiung Medical University Hospital, Kaohsiung 807, Taiwan; 5 Division of Hematology-Oncology, Kaohsiung Medical University Hospital, Kaohsiung 807, Taiwan; 6 Department of Pathology, Kaohsiung Medical University Hospital, Kaohsiung 807, Taiwan; 7 Simmons Comprehensive Cancer Center, Departments of Pharmacology and Biochemistry, University of Texas Southwestern Medical Center, Dallas, TX 75390-8807 USA; 8 Division of Environmental Health and Occupational Medicine, National Health Research Institutes, 115, Zhunan, Taiwan; 9 Center of Infectious Disease and Cancer Research, Kaohsiung Medical University, Kaohsiung 807, Taiwan; 10 Center for Stem Cell Research, Kaohsiung Medical University, Kaohsiung 807, Taiwan; 11 Department of Molecular Preventive Medicine, Graduate School of Medicine, the University of Tokyo, Tokyo, 113-0033 Japan; 12 Department of Medical Research, Kaohsiung Medical University Hospital, Kaohsiung Medical University, Kaohsiung 807, Taiwan

**Keywords:** Cancer genetics, Cell biology

## Abstract

Metastasis is the main cause of cancer mortality. However, the triggering mechanisms and regulation of epithelial–mesenchymal transition (EMT) factors in the commitment of metastasis have not been well characterized. Spermatogenic Zip 1 (SPZ1) acts as a proto-oncogene and an upstream regulator of EMT during tumorigenesis. Here we report that the HIV-1 Tat-interacting protein 60 kDa (Tip60) acetyltransferase mediates acetylation at lysine residues of SPZ1 at positions 369 and 374, and of TWIST1 at positions 73 and 76, which are required for SPZ1–TWIST1 complex formation and cancer cell migration in vitro and in vivo. Ectopic SPZ1 and TWIST1 expression, but not that of TWIST1 alone, enhanced vascular endothelial growth factor (VEGF) expression via the recruitment of bromodomain-containing protein 4 (BRD4), thus enhancing RNA-Pol II-dependent transcription and inducing metastasis. Neutralization of VEGF using humanized monoclonal antibodies such as Avastin, effectively abrogated the EMT and oncogenesis induced by the acetylated SPZ1–TWIST1 complex. Our findings highlight the importance of acetylation signaling in the SPZ1–TWIST1–BRD4 axis in the mediation of EMT and its regulation during tumor initiation and metastasis.

## Introduction

Metastasis, a leading cause of poor prognosis in patients with cancer, accounts for 90% of all cancer-related deaths. Despite the identification of potential oncogenic drivers and their roles as master regulators of cancer initiation, the mechanisms underlying tumorigenesis and metastasis remain unclear [1]. The epithelial–mesenchymal transition (EMT) is a critical process in metastasis and oncogenesis [2].

TWIST1, a basic helix-loop-helix (bHLH) transcription factor, was originally identified as a mesoderm-inducing factor in *Drosophila* [3] and is known as a major inducer of EMT in human mammary epithelial cells [4] and other cancers such as sarcoma, melanoma, and lymphoma [4, 5]. Increased TWIST1 expression promotes EMT by regulating cell motility and invasive activity and enhances some features of cancer stem cells through control of downstream gene expression [5, 6]. One unique function of TWIST1 is that it represses the transcription of the E-cadherin promoter via *cis*-elements [7] and p53 expression controlled indirectly via p14^Arf^ and the mouse double minute 2 homolog [8]. WNT signaling can be affected by TWIST1 via the inhibition of the coactivator p300/CBP and other mechanisms [9]. However, the molecular pathways through which TWIST1 and the downstream events are regulated during tumor metastasis have not been well characterized [4].

The spermatogenic leucine zipper protein 1 (*SPZ1*), a bHLH–ZIP transcription factor, is expressed primarily during embryonic development, including in stem cells and in early gastrulation stages; however, it shows a testis-specific expression pattern in germline and somatic cells (Sertoli and Leydig cells) in the adult stage [10]. Mitogen-activated protein kinase signaling promotes SPZ1 expression and activation by phosphorylation, resulting in SPZ1 translocation into the nucleus and activation of downstream gene expression such as that of the proliferating cell nuclear antigen [11]. The DNA-binding elements recognized by SPZ1 on its target promoters include consensus sequence E-boxes (CANNTG or CACGTG) or the variant sequence G-box-like motif (GGG/AGGGG/AA/TT) [11]. Functionally, ectopic SPZ1 was shown to promote cell-cycle progression and proliferation and induce carcinogenesis, both in vitro and in vivo, including the formation of liver tumors in transgenic mice [11, 12]. SPZ1 overexpression has been reported in several human tumors [11]. Moreover, SPZ1 was shown to mediate EMT signaling and regulate tumor metastasis by transactivation of *TWIST1* expression [13]. Despite the potential oncogenic activity of SPZ1, the detailed regulatory mechanisms of SPZ1 remain unclear.

We show here that (1) TIP60 acetylates SPZ1 and TWIST1, (2) acetylated SPZ1 interacts with acetylated TWST1, and (3) this complex recruits the bromodomain-containing protein 4 (BRD4) to enhance RNA polymerase II (Pol II) transcription [14], thereby promoting angiogenesis and metastasis in vitro and in vivo. Therefore, SPZ1 is an important regulator of tumor metastasis and cell plasticity in the tumorigenic microenvironment.

## Results

### SPZ1 directly interacts with TWIST1 in vitro and in vivo

**Fig. 1:**
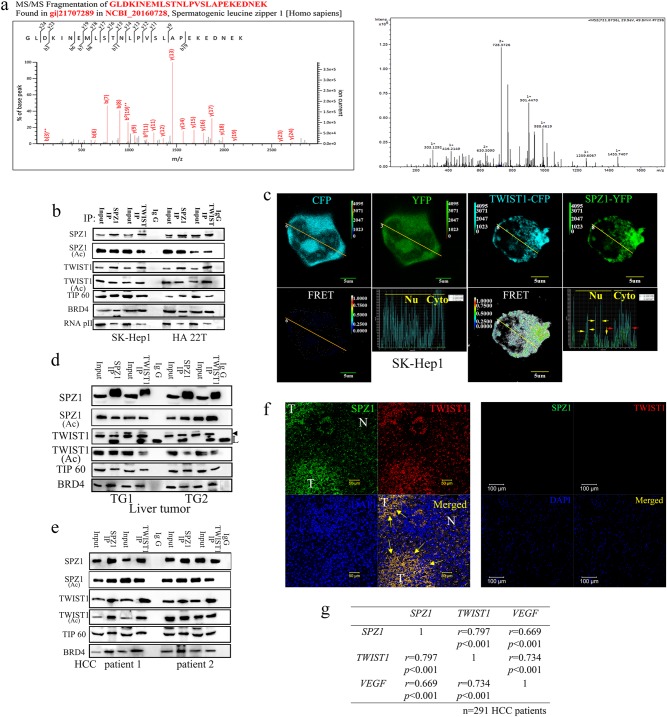
SPZ1 interacts with TWIST1 in vitro and in vivo. **a** The SPZ1 protein was detected in anti-TWIST1 immunoprecipitates. The SPZ1 protein (No. 358 in Fig. S1a) obtained from anti-TWIST1 immunoprecipitates of SK-Hep1 cell lysates was identified by liquid chromatography‒tandem mass spectrometry (LC-MS-MS). **b** SPZ1-GFP associates with FLAG-TWIST1 and its interaction with other proteins (TIP60, BRD4, and Pol II) in SK-Hep1 and HA 22T cells, as assayed by immunoprecipitation (IP) and western blotting. **c** SPZ1-YFP colocalized with TWIST1-CFP in SK-Hep1 cells, as determined by fluorescence resonance energy transfer (FRET) assay. Green, YFP; cyan, CFP; FRET signals (lower panels). The oblique line indicates the analyzing sites for FRET. The red and yellow arrows indicate cytosol and nuclei, respectively. **d** SPZ1 interacts with TWIST1 in liver tumors from *Spz1* transgenic mice, TG1 and TG2. L: light chain; arrowhead, TWIST1. **e** SPZ1 interacts with TWIST1 in tumor tissues derived from patients with HCC. **f** Colocalization of SPZ1 and SPZ1 in HCC tumor samples. Green, SPZ1; red, TWIST1; and blue (DAPI), nuclei. T HCC tumor, N normal liver cells. Yellow arrow: SPZ1-TWIST1 complex in tumor cells of HCC. **g** mRNA expression of *SPZ1* in paired-HCC tumor samples (normal vs. tumor tissues) correlates significantly with the mRNA expression of *TWIST1* and *VEGF*. Correlations were assessed using Pearson’s correlation coefficient (*r*)

Epithelial–mesenchymal transition (EMT) has been proposed as a key step in tumor progression and metastasis. The hallmark of EMT is loss of epithelial marker expression (E-cadherin and catenin) and gain of mesenchymal markers (N-cadherin, Vimentin, and SMS-actin). TWIST1 has been implicated in tumor initiation, stemness, angiogenesis, dissemination, and chemoresistance in various carcinomas, sarcomas, and hematological malignancies [15]. However, the precise targets of, or molecules associated with, TWIST1 have not been well characterized, with the exception of MEF2 [16], TCF3, p300/PCAF [17], and its interaction with BRD4 [18]. To elucidate the potential regulatory mechanisms of TWIST1 signaling in tumorigenesis and metastasis, co-immunoprecipitation coupled with two-dimensional gel electrophoresis (2-DE) and liquid chromatography–mass spectrometry was conducted to identify TWIST1-interacting proteins in lysates of the aggressive hepatoma cell line SK-Hep1 (Fig. 1a). This approach yielded six candidate proteins from three independent 2-DE experiments (Supplementary Figure S1a). The oligopeptides GLDKINEMLSTNLPVSLAPEKEDNEK (amino acids 115‒140) and SQKDISETCGNNGVGFQTQPNNEVSAK (amino acids 226‒252) were detected via liquid chromatography–mass spectrometry, sequenced, and their origin identified as SPZ1 (gi 21707289) (Fig. 1a, Supplementary Fig. S1a, and S1b). The expression levels of SPZ1 were previously shown to be higher in the aggressive hepatoma cell lines SK-Hep1 and HA 22T than in HepG2 and Huh 7 hepatoma cells, while the Alexander hepatoma cell line PLC5, Hep 3B, and benign hepatocytes (Chang liver CNL) had lower or undetectable expression of this protein [13].

To confirm the interaction between SPZ1 and TWIST1 in vitro, immunoprecipitates of SPZ1 and TWIST1 from hepatoma cells were examined by immunoblotting. Endogenous TWIST1 was detected in SPZ1 immunoprecipitates, and endogenous SPZ1 was detected in TWIST1 immunoprecipitates (Fig. 1b). Interestingly, the newly identified TWIST1-interacting proteins including BRD4, TIP60, and Pol II [18] were also detected in both SPZ1 and TWIST1 immunoprecipitates. Several known epigenetic TWIST-interacting proteins such as CREB, CBP, and MyoD, were detected in anti-TWIST1 immunoprecipitates, but not in anti-SPZ1 immunoprecipitates (data not shown).

These results were further confirmed by confocal fluorescence resonance energy transfer (FRET) analysis, in which yellow fluorescent protein (YFP)-tagged SPZ1 (SPZ1-YFP; green) and cyan fluorescent protein-tagged TWIST1 (TWIST1-CFP; cyan) were found to colocalize (white FRET signal) primarily in the cytoplasm and to a lesser extent, in the nuclei of SK-Hep1 (Fig. 1c) and Huh 7 cells (Supplementary Figure S2a). The interaction between SPZ1 and TWIST1 was further evaluated in tumors and adjacent healthy liver tissues from *SPZ1* transgenic mice and patients with hepatocellular carcinoma (HCC). A significant amount of TWIST1 was detected in SPZ1 immunoprecipitates of liver tumor tissues from *SPZ1* transgenic mice and patients with HCC. Reciprocally, SPZ1 was also detected in TWIST1 immunoprecipitates of the samples. Morevoer, acetylated SPZ1 and acetylated TWIST1 were also detected in reciprocal immunoprecipitates (Fig. 1d, e). In contrast, little or no TWIST1 was detected in SPZ1 immunoprecipitates of adjacent healthy liver tissues from transgenic mice or from HCC patients, whereas low levels of SPZ1 were detected in TWIST1 immunoprecipitates of the samples. Acetylated TWIST1 was not detected in SPZ1 or TWIST1 immunoprecipitates of adjacent healthy liver tissues (Supplementary Figure S2b). Confocal fluorescence imaging was used to examine the interaction between SPZ1 and TWIST1 in immunostained samples of tumor masses and adjacent healthy liver tissues from patients with HCC, showing TWIST1 protein expression (red) and SPZ1 expression (green) in HCC cells (Fig. 1f). To explore the relationship between SPZ1, TWIST1, and vascular endothelial growth factor (VEGF) in HCC, expression patterns of the *SPZ1*, *TWIST1*, and *VEGF* mRNAs were examined in HCC tumor samples from 291 patients with HCC. *SPZ1* mRNA expression correlated strongly with *TWIST1* and *VEGF* mRNA expression in these patients (Pearson correlation coefficients, *r* = 0.797 and 0.669, respectively, *P* < 0.0001; Fig. 1g). Therefore, SPZ1 appeared to interact with TWIST1 in cultured hepatoma cells, *SPZ1* transgenic mice, and HCC tumor specimens.

### The bHLH domain of SPZ1 interacts with the WR domain of TWIST1

Coimmunoprecipitation was used to identify the interaction domain of SPZ1 that associates with TWIST1 in Huh 7 cells. GFP-tagged SPZ1 and the deletion mutants SPZ1ΔB (SPZ1 with a bHLH domain deletion) and SPZ1ΔL (SPZ1 with a leucine zipper deletion), were transiently expressed and subsequently immunoprecipitated using an anti-GFP antibody (Supplementary Figure S3a). HA-tagged TWIST1 showed high-affinity binding to the full-length and truncated SPZ1 proteins, but not to SPZ1ΔB (left panel in Supplementary Figure S3a). SPZ1 and SPZ1ΔL also showed high-affinity binding to TWIST1 (right panel in Supplementary Figure S3a). Similarly, mCherry-tagged TWIST1 and the truncated proteins showed high-affinity binding to GFP-tagged SPZ1, but not to TWIST1 with a WR domain deletion (TWIST1ΔWR) (Figure S3b).

To understand how the interaction between SPZ1 and TWIST1 increases cell proliferation and expression of metastasis-related targets, we co-expressed them in the cells and examined cell proliferation and the expression of metastasis-related proteins. Hep 3B cells co-expressing TWIST1 and SPZ1 showed significantly greater upregulation of proliferation and EMT markers compared to cells expressing the individual proteins (Supplementary Figure S3c, S3d, and S3e). Further, SK-Hep1 cells co-expressing SPZ1 and full-length TWIST1 or TWIST1ΔB but not those co-expressing SPZ1 and TWIST1ΔWR, exhibited EMT-related protein expression (Supplementary Figure S3e). Conversely, Hep 3B cells co-expressing TWIST1 and full-length SPZ1 or SPZ1ΔL but not those co-expressing TWIST1 and SPZ1ΔB, showed increased EMT-related protein expression compared to cells expressing the individual proteins (Supplementary Figure S3d). Hep 3B cells co-expressing SPZ1ΔB and TWIST1 showed no changes in EMT-related protein expression and lesser inhibition of cell proliferation and EMT marker expression compared to cells expressing the individual proteins (Supplementary Figure S3c and S3d).

**Fig. 2:**
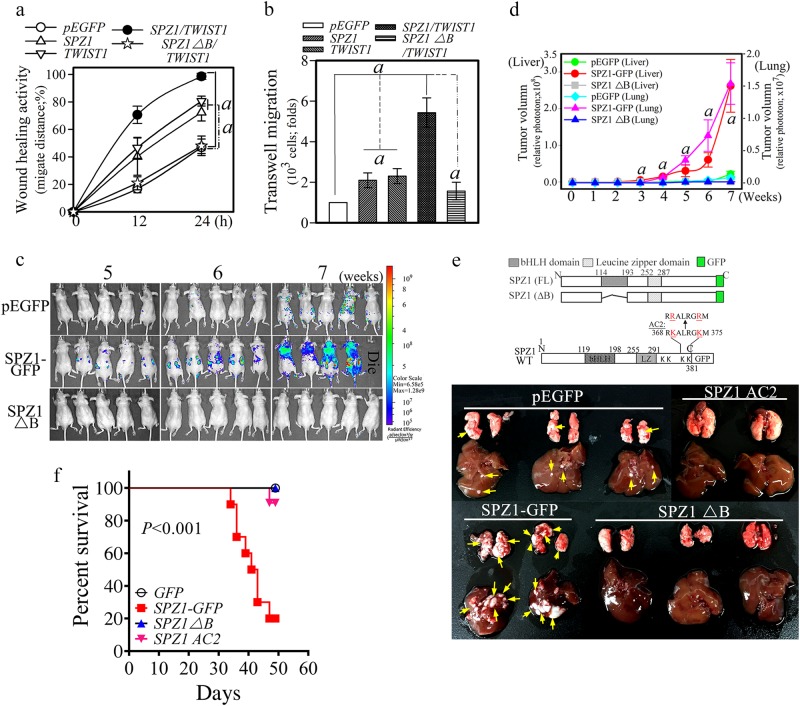
SPZ1-TWIST1 interaction regulates tumor metastasis. The results are shown as the mean ± s.d. *a*, *P* < 0.001. Each experiment was repeated at least three times. **a** Coexpression of genes encoding *SPZ1-GFP* and *TWIST1* promotes wound-healing activity in Huh 7 cells. **b** Coexpression of *SPZ1-GFP* and *TWIST1* promotes invasive growth of Huh 7 cells. **c** Oncogenic potential of *SPZ1-GFP*, but not SPZ1ΔB, in xenografts derived from SK-Hep1 cells. SK-Hep 1 cells constitutively expressing RFP were transfected with various constructs and directly injected into livers. Tumor growth and metastasis were monitored using an IVIS imaging system, as described in the “Materials and Methods” section. **d** *SPZ1-GFP-* induced tumor growth activity in the liver and lung. (*n* = 10). **e** Tumor formation in nude mice injected with SK-Hep1 cells carrying *SPZ1-GFP* constructs. Yellow arrows, tumor mass. **f** Survival curve analysis of nude mice injected with SK-Hep1 cells carrying SPZ1-GFP constructs (*n* = 10)

The regulatory effects of the SPZ1–TWIST1 complex on EMT were examined by assessing wound-healing and cell-migration activity. In assays of wound-healing and transwell migration, Huh 7 cells expressing either SPZ1 or TWIST1 exhibited greater cell mobility than mock-transfected control cells, whereas cells expressing both SPZ1 and TWIST1 exhibited an additive effect of wound-healing and cell motility. In contrast, Huh 7 cells co-expressing TWIST1 and SPZ1ΔB exhibited no wound-healing or cell-migration activities (Fig. 2a, b). Therefore, the bHLH domain of SPZ1 appears to be critical for its functional interaction with TWIST1. To replicate these findings in the context of cancer development and metastasis, we used IVIS imaging to evaluate tumor cell growth and metastasis in nude mice injected (via tail vein) with constitutively RFP-expressing SK-Hep1 cells expressing pEGFP, SPZ1-GFP, or SPZ1ΔB. Imaging of SK-Hep1-SPZ1-GFP cells was first performed at 5 weeks post-injection, and larger images were captured at seven weeks post-injection (Fig. 2c). Tumors in livers and lungs were detected beginning at the fifth week after injection of RFP-SK-Hep1-SPZ1-GFP cells. However, SK-Hep1 cells expressing pEGFP alone or mutant SPZ1ΔB showed minor or no primary tumor formation in the lung (Fig. 2d). Nude mice injected with SK-Hep1 cells expressing SPZ1-GFP, but not those injected with cells expressing pEGFP or SPZ1ΔB, showed limited survival and died 7–8 weeks after injection (Fig. 2f). The liver and lung tissues of each nude mouse were also examined for tumor growth via IVIS imaging (Fig. 2e). Thus, we found that the B domain (bHLH domain) of SPZ1 may be critical for tumor growth, invasion, and metastasis in vivo, possibly through interaction with TWIST1. In particular, the lysine residues K369 and K374 in SPZ1 were found to be critical for tumor growth, and this was confirmed using the AC mutant of SPZ1 (Fig. 2e).

### The SPZ1–TWIST1 complex transcriptionally activates human *VEGF* gene expression

**Fig. 3:**
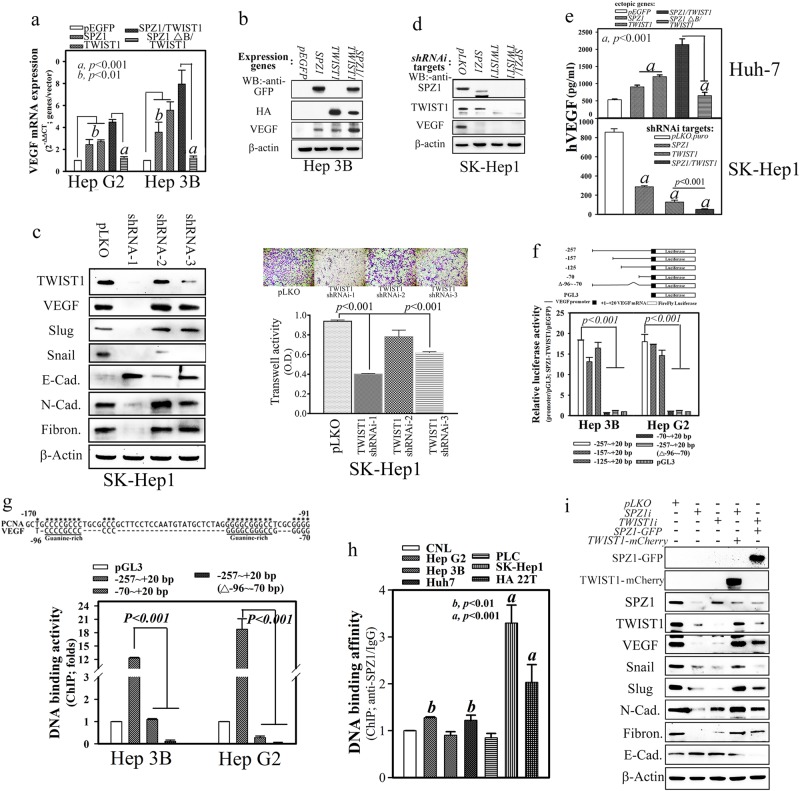
Coexpression of *SPZ1-EGP* and *HA-TWIST1* transactivates the *VEGF* promoter in hepatoma cells. Results were expressed as the mean ± s. d. *a*. *P* < 0.001; *b*. *P* < 0.01. Each experiment was repeated at least three times. **a** Hepatoma cells (Hep G2 and Hep 3B) transfected with *SPZ1-GFP* and/or *HA-TWIST1*, but not those transfected with *SPZ1ΔB-GFP*, showed increased expression of the *VEGF* mRNA. **b** Effects of forced expression of *SPZ1-GFP* and *HA-TWIST1* on VEGF expression in Hep 3B cells, respectively. **c** Effects of three shRNA constructs (shRNA-1, -2, and -3) on expression of TWIST1 in SH-Hep1 cells. Three shRNAi-TWIST1 recombinant viruses and pLKO control virus were prepared and transfected in cells of 6-well culture plates and incubated at 37 °C. Twenty-four hours later, the conditioned medium was collected and centrifuged at 3000 r.p.m. for 20 min to remove cell debris. The supernatants were then subjected to western blotting and transwell assay as described in Materials and Methods. **d** Effects of shRNA against SPZ1 and TWIST1 and coexpression of both shRNAi-SPZ1 and shRNAi- TWIST1 on expression in SK-Hep1 cells, respectively. (**e**) Coexpression of *SPZ1-GFP* and *Flag-TWIST1* in Huh 7 cells significantly enhances the production of VEGF in the culture medium compared with that observed in *SPZ1-GFP-* or vector-transfected Huh 7 cells; however, VEGF expression in SK-Hep1 cells was reduced significantly by the introduction of knockdown constructs of *shRNAi-SPZ1* and *shRNAi-TWIST1*. Results were expressed as the mean ± s.d. *a*, *P* < 0.001. **f** Schematic representation of a series of *VEGF* promoter-luciferase deletion constructs. Relative luciferase activity in Hep 3B and Hep G2 cells was measured as described in “Materials and Methods” section. **g** Transactivation induced by coexpression of *SPZ1-GFP* and *HA-TWIST1* on *VEGF* promoter-luciferase. The region located between ‒96 and ‒76 bp, is critical in Hep G2 and Hep 3B cells. **h** ChIP assay of the *VEGF* promoter in various hepatoma lines using anti-SPZ1 antibodies described in the “Materials and Methods” section. SK-Hep1 and HA22T, high expressions of SPZ1; Hep G2 and Huh 7, low expression of SPZ1; Hep 3B and PLC/PRF/5, medium SPZ1 expression. **i** Effect of *TWIST1* shRNAi in SK-Hep1 cells carrying Tet-ON inducible *SPZ1-GFP* on various mesenchymal marker proteins. CNL Chang normal liver cells

To ascertain whether VEGF (an angiogenic marker) was indeed activated by the SPZ1–TWIST1 complex [13], Hep 3B cells, which show low expression of SPZ1 and TWIST1, were transfected with cDNA constructs encoding SPZ1 or TWIST1 separately; the cells showed an apparent increase in *VEGF* mRNA and protein expression (2.81- and 3.01-fold, respectively in Fig. 3a, b). In comparison, Hep 3B cells co-expressing SPZ1 and TWIST1 showed a greater increase in VEGF expression. In contrast, Hep G2 and Hep 3B cells co-expressing SPZ1∆B and TWIST1 showed no such increases in VEGF expression (Fig. 3a). To evaluate the essential effect of SPZ1 and TWIST1 on VEGF expression further, three sequence-specific short hairpin RNAs (shRNAs; e.g., lentivirus shRNA-1, -2, and -3) against TWIST1 were introduced into SK-Hep1 cells. Expression of TWIST protein was efficiently suppressed by 95% using shRNA-1 and by ~50% using shRNA-3. In addition, the expression of EMT-related proteins was also suppressed and that of E-cadherin was enhanced (Fig. 3c). However, shRNA-2 was not functional. Similarly, the migration/invasion activity of cells transfected with shRNA-1 and shRNA-3 reduced by 95 and 50%, respectively, but did not decrease in those transfected with shRNA-2 (Fig. 3c). Thus, shRNA-1 construct (designated as TWIST1i) was used in further experiments. SK-Hep1 cells transfected with SPZ1 [19] or TWIST1 shRNA showed significantly decreased VEGF expression (Fig. 3d). The level of VEGF in the culture medium was increased when Huh 7 cells were transfected with cDNAs encoding either SPZ1 or TWIST1, and increased further in co-transfected cells (Fig. 3e, upper panel). However, co-transfection of Huh 7 cells with cDNAs encoding SPZ1∆B and TWIST1 did not lead to a significant increase in VEGF levels in the culture medium (Fig. 3e, upper panel). Conversely, knockdown of SPZ1 and/or TWIST1 in SK-Hep cells led to a marked decrease in VEGF protein levels in the culture medium (Fig. 3e, lower panel).

To identify the potential transactivation mechanism underlying the effect of the SPZ1–TWIST1 complex on *VEGF* expression, serially truncated *VEGF* promoter regions were subcloned to create luciferase cDNA expression constructs and co-transfected with those expressing SPZ1 and TWIST1 into hepatoma cells. The SPZ1–TWIST1 complex significantly increased luciferase activity (Hep 3B, 18.35-fold; and Hep G2, 18.61-fold) when the repoter was driven by the *VEGF* promoter encompassing the −70 bp region (‒257 to +20 bp) or by the *VEGF* promoter lacking the region between −96 and −70 bp (−257 to +20∆–96 to −70) (Fig. 3f); however, promoter recruitment at the −257 to +20∆–96 to −70 fragment was significantly reduced by antibodies against SPZ1 and TWIST1 in chromatin immunoprecipitation (ChIP) assays (Fig. 3g). ChIP analysis was used to evaluate chromatin binding of the SPZ1–TWIST1 complex to the endogenous *VEGF* promoter. Aggressive hepatoma cells (SK-Hep1 and HA 22T), which express high levels of the VEGF protein, showed enhanced recruitment of the SPZ1–TWIST1 complex to the *VEGF* promoter compared with that observed in cells with low or no VEGF (Hep 3B and PLC, respectively) expression (Fig. 3h).

The effect of the SPZ1–TWIST1 complex on the regulation of EMT-related genes was evaluated in SK-Hep1 cells. Rescue experiments of TWIST1-mCherry on SPZ1 shRNAi showed the recovery of EMT-related proteins. Moreover, the rescue experiments of SPZ1-GFP on TWIST1-mCherry also showed the recovery of EMT-related proteins (Fig. 3i).

The expression of VEGF and that of the endothelial marker CD31 was determined in tumor tissues injected with SK-Hep1 cells expressing GFP-tagged SPZ1. VEGF expression was colocalized with ectopic SPZ1 expression in the tumor tissues, in contrast with the observations using mock-transfected or *SPZ1* shRNA-transfected SK-Hep1 cells (Supplementary Figure S4a). Moreover, in liver tumors of *SPZ1* transgenic mice, perivascular colocalization of SPZ1 with high VEGF expression was also observed (Supplementary Figure S4b). Therefore, the SPZ1–TWIST1 complex appears to regulate *VEGF* expression and secretion, which consequently regulates angiogenesis in tumor masses. The endothelial marker CD31 was also colocalized with SPZ1 expression in liver tumors of *SPZ1* transgenic mice. Higher CD31 expression was detected in a radial configuration in perivascular regions of tumor tissues injected with SK-Hep1 cells; conversely, tumor masses injected with *SPZ1* shRNA-transfected SK-Hep1 cells showed a lower level and a more scattered pattern of CD31 expression (Supplementary Figure S4c and S4d).

### Acetylation of the SPZ1–TWIST1 complex by TIP60 is essential for cell proliferation and EMT induction

**Fig. 4:**
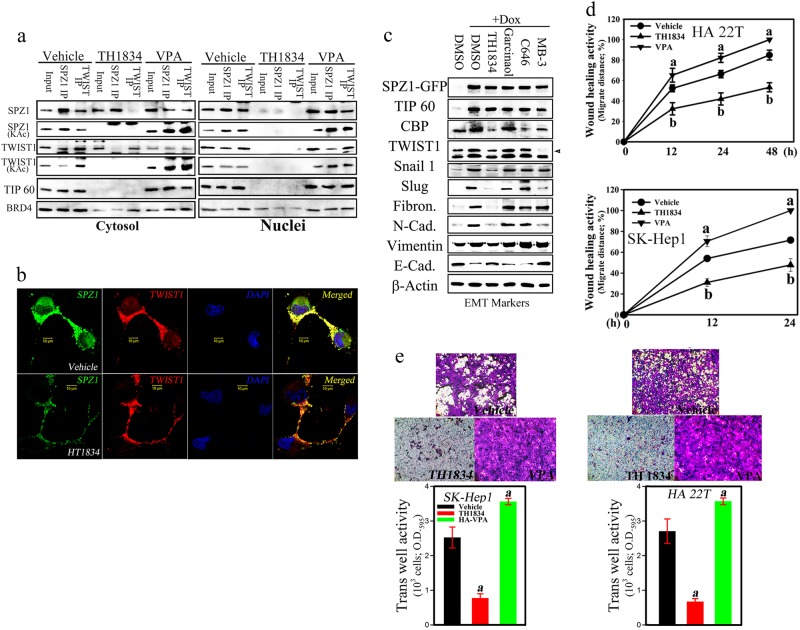
Effect of an inhibitor of the acetylation of TIP60 on the SPZ1-TWIST1 complex and wound-healing and invasive activity. Results are presented as the mean ± s. d. a, *P* < 0.001 *b, P* < 0.01. Each experiment was repeated at least three times. **a** The TIP60 inhibitor, TH1834 (20 μM), blocks lysine acetylation and the interaction between SPZ1 and TWIST1 in nuclei and the cytoplasm of SK-Hep1 cells. **b** TH1834 blocks nuclear translocation of SPZ1 and TWIST1 in SK-Hep1 cells. Green, SPZ1-GFP; red, TWIST1-Flag; blue, DAPI. **c** TH1834 abolishes the expression of EMT marker proteins in SK-Hep1 cells induced by Dox. **d**, **e** Hepatoma cells treated with the TIP60 inhibitor exhibites inhibition of wound-healing activity (Supplementary Figure S5f; d) and invasive growth. **e** Coexpression of *SPZ1-GFP* and *Flag-TWIST1* in SK-Hep1 and HA 22T cells

Considering the fact that diacetylated TWIST1 induced by TIP60 recruits BRD4 which is associated with acetylated H3 and H4 histones, as well as P-TEFb of the RNA-Pol II elongation complex at the promoter and enhancer regions of target genes such as *WNT5A* [18], we next investigated the mechanisms underlying the formation of the SPZ1–TWIST1 complex. An acetylation inhibitor selective for TIP60, TH1834 (20 μM), inhibited the acetylation of SPZ1 and TWIST1 in the SPZ1-TWIST1 complex in cytoplasmic and nuclear fractions (Fig. 4a, b, and Supplementary Figure S5a). We used four acetyltransferase inhibitors to determine whether the acetylation of SPZ1 is essential for EMT induction (Fig. 4c) and SPZ1–TWIST1 complex formation (Fig. 4b, c, and S5b). The results showed that TH1834 (20 μM) suppressed the expression of TIP60 and proliferation-related genes in SK-Hep1 and HA 22T cells, and enhanced EMT marker expression induced by ectopic SPZ1 expression (Supplementary Figure S5c and S5d). A general histone deacetylase inhibitor, valproic acid (VPA; 1 mM), had the opposite effect of upregulating SPZ1 and TWIST acetylation (Fig. 4a, S5a, and Supplementary Figure S5b). Inhibition of both SPZ1 and TWIST1 acetylation induced by TH1834 treatment also abolished the transactivation and binding of SPZ1 to the VEGF promoter (Supplementary Figure S5e). SK-Hep1 and HA 22T cells treated with TH1834 showed significantly decreased migration activity, as determined using wound-healing (Fig. 4d and Supplementary Figure S5f) and transwell migration assays (Fig. 4e). TH1834 also markedly decreased the expression of several proliferation markers (Cyclin D1, Ki67, and E2F1) (Supplementary Figure S5c) and EMT/MET markers (Slug, E-cad, N-cad, Fibronectin, and Vimentin) (Supplementary Figure S5d).

### Acetylation of TWIST1 lysine^73^ and lysine^76^ by TIP60 is essential for the cellular function of the SPZ1–TWIST1 complex

**Fig. 5:**
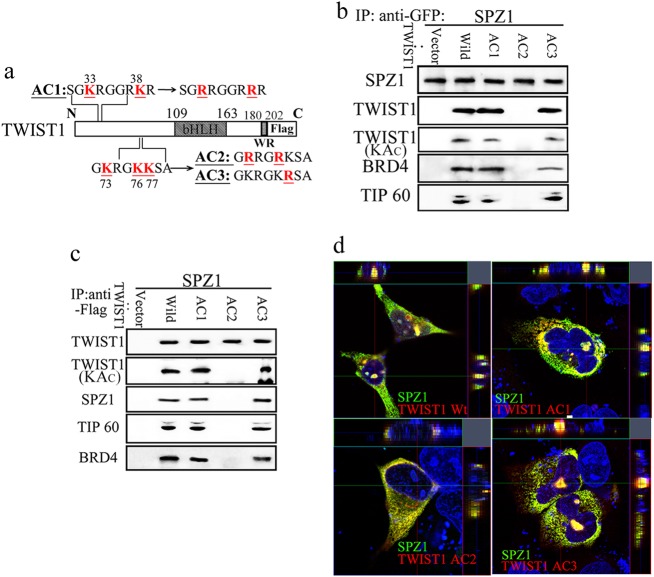
Acetylation of lysines residues at 73 and 76 of TWIST1 is critical for the SPZ1-TWIST1 association. **a** Schematic representation of the TWIST1 domain organization and its lysine mutants AC1‒AC3. **b**, **c** SPZ1-GFP (b) and FLAG–SPZ1 (c) interact with wild-type, AC1, and AC3, but not AC2, to form the functional complex of SPZ1/TWIST1/BRD4 in SK-Hep1 cells. **d** SPZ1 GFP does not interact with the AC2 mutant in the nuclei of SK-Hep1 cells. Green, SPZ1-GFP; red, TWIST1 mutants; blue, DAPI

Potential acetylation sites on TWIST1 were determined and point mutations were created to examine the necessity of acetylation induced by TIP60 on SPZ1–TWIST1 complex formation. SK-Hep1 cells co-expressing SPZ1 and each TWIST1 mutant, except for the AC2 mutant (K73R/K76R) [18], showed interaction of the proteins with each other and increased expression of proliferation and EMT markers (Fig. 5a and S6a). Further, the TWIST1 AC2 mutant (K73R/K76R) showed no binding with SPZ1 and TIP60 in forming a transcription complex that interacts with BRD4 in SK-Hep1 cells (Fig. 5b, c, and Supplementary Figure S6b). SK-Hep1 and Huh-7 cells co-expressing SPZ1 and TWIST1 AC2 showed a lack of SPZ1–TWIST1 complex (yellow) in the nuclei (Fig. 5d and Supplementary Figure S6c), and no recruitment to the VEGF promoter (Supplementary Figure S6d and S6e). This might have been reflected in the lack of VEGF protein in the culture medium (Supplementary Figure S6f).

### Acetylation of SPZ1 lysine^369^ and lysine^374^ by TIP60 is essential for the cellular function of the SPZ1–TWIST1 complex

**Fig. 6:**
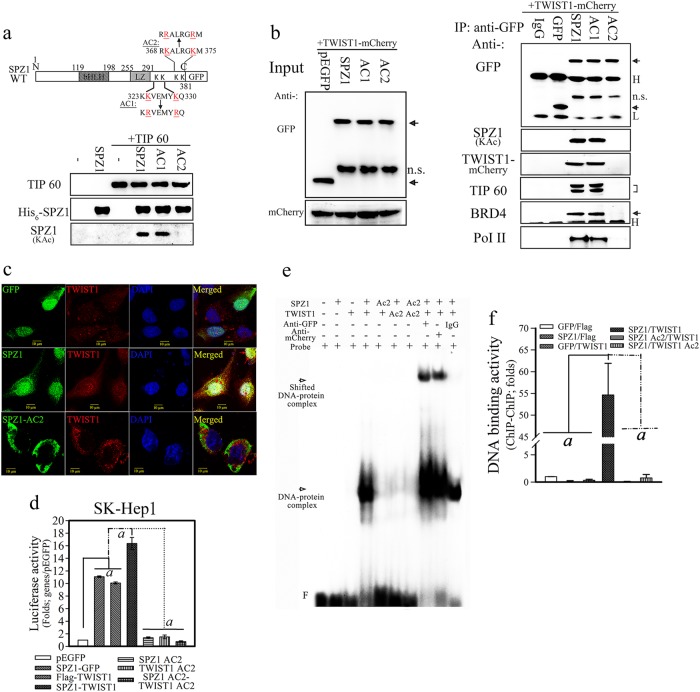
Acetylation of lysine residues 369 and 374 of SPZ1 is essential for SPZ1-TWIST1 complex formation. **a** Schematic representation of the SPZ1 domain structure and its mutants AC1 and AC2. Recombinant TIP 60 acetylates His_6_-SPZ1 at lysine residues 369 and 374. **b** TWIST1-mCherry interacts with acetylated SPZ1-GFP in vivo. n.s. indicates nonspecific interaction. The SPZ1-GFP mutant, AC2, does not interact with TWIST1-mCherry, TIP60, BRD4, and Pol II. L, light chain; H, heavy chain; arrow, SPZ1-GFP. **c** Mutation at the acetylation site of SPZ1 (AC2) abolishes the interaction and nuclear translocation of the SPZ1–TWIST1 complex. Green, SPZ1; red, TWIST1; blue, DAPI. **d** Forced expression of both SPZ1-GFP AC2 and/or TWIST1 AC2 or both constructs abolishes VEGF promoter-luciferase activity in SK-Hep1 and Huh 7 cells (data not shown). *a, p* < 0.001. **e** Supershift EMSA with recombinant wild-type SPZ1-GFP, mutant SPZ1-GFP AC2, mCherry-TWIST1, and TWIST1-mCherry AC2 proteins using the G-rich sequence (‒96 to ‒70 bp) DNA probe o from human VEGF promoter in the presence of anti-GFP or anti-mCherry or control IgG. Arrows indicate the DNA–protein complex and shifted DNA–protein complex (F, free DNA probe). **f** ChIP-ChIP assay of chromatin samples of transformed SK-Hep1cells expressing wild-type SPZ1 and TWIST1 and their variants in SK-Hep1 cells. *a, P* < 0.001

Potential acetylation sites on SPZ1 were determined and point mutations were created to examine their specific cellular effects on the SPZ1–TWIST1 complex. His_6_-tagged wild-type SPZ1 and mutants were incubated with active recombinant TIP60 to evaluate the potential acetylation sites in vitro. Wild-type SPZ1 and the SPZ1 AC1 mutant but not the AC2 mutant (K369/K374A) were acetylated by TIP60, indicating that the lysine residues at positions 369 and 374 in SPZ1 are substrates of TIP60 in vitro (Fig. 6a). In SK-Hep1 cells co-expressing TWIST1 and SPZ1 mutants except for the AC2 mutant, the SPZ1–TWIST1 complex was detected in anti-GFP immunoprecipitates, suggesting that acetylation at lysine residues 369 and 374 in SPZ1 is critical for complex formation with BRD4 and TIP60 in vivo (Fig. 6b). Neither SPZ1 nor the SPZ1–TWIST1 complex was detected in nuclei of SK-Hep1 cells expressing SPZ1 AC2, even though some TWIST1 was detected in the nuclei (Fig. 6c).

Our finding suggests that lysine residues 369 and 374 in SPZ1 and lysine residues 73 and 76 in TWIST1 were critical for the mutual interaction of the proteins, although their direct interaction domains were located outside the regions containing these lysine residues. To examine the mechanism further, combinations of these mutants on VEGF promoter activity were studied. Neither individual mutants of SPZ1 and TWIST1 nor a combination of the mutants resulted in increased VEGF promoter activity (Fig. 6d). Further, to investigate DNA contact of the TWIST1/SPZ1 complex in vitro and in vivo, two-step chromatin immunoprecipitation (ChIP-ChIP) and electrophoretic mobility shift assay (EMSA) were performed. Recombinant proteins such as SPZ1-GFP and TWIST1-mCherry were prepared, and a EMSA was performed using [^32^P]-labeled G-rich sequence probe corresponding to the region of −96 to −70 bp derived from the human VEGF promoter (Fig. 6e). A DNA–protein complex was detected in samples with a combination of recombinant SPZ-1 and TWIST1 proteins but not in samples with either of the proteins alone. In addition, a prominent supershift was detected in samples treated with antibodies against GFP and mCherry but not in those treated with control IgG. Moreover, the DNA–protein complexes derived from either SPZ1-GFP AC2 or TWIST1-mCherry AC2-purified protein were not detected on gels (Fig. 6e). Furthermore, a two-step ChIP-chip analysis revealed a clear recruitment of SPZ1 and TWIST to chromatin from SPZ1- and TWIST1-expressing cells (Fig. 6f) but not from cells expressing AC2 mutants of SPZ1 or TWIST1 (lanes 5 and 6 in Fig. 6f).

Both SK-Hep1 and Huh 7 cells expressing wild-type SPZ1 or AC1, but not AC2, exhibited activated EMT-marker expression (Supplementary Figure S7a). SK-Hep1 cells expressing the SPZ1 AC2 mutant showed significantly decreased migration activity (Supplementary Figure S7b and S7c) compared with the cells expressing wild-type SPZ1 or AC1.

### A VEGF-neutralizing antibody, Avastin, significantly suppresses SPZ1–TWIST1 complex-induced cell proliferation and metastasis of hepatoma cells in vitro and in vivo

**Fig. 7:**
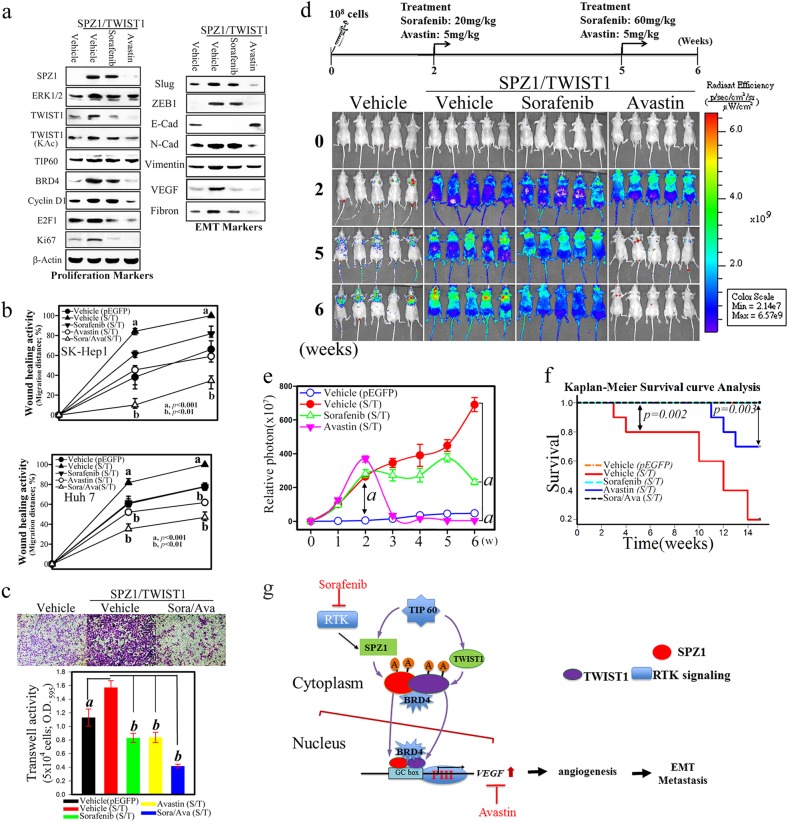
Monoclonal antibody against Avastin suppresses VEGF expression and inhibits metastasis induced by SPZ1 and TWIST1 coexpression in SK-Hep1 cells. Results are presented as the mean ± s. d. a, *P* < 0.001 and b, *P* < 0.01. Each experiment was repeated at least three times. **a** A low dose of Avastin (1.25 ng/mL), compared with a low dose of the RTK inhibitor (Sorafenib; 10 μM), abolished the expression of VEGF and other EMT markers in Huh 7 cells induced by *SPZ1-GFP* and *FLAG-TWIST1* coexpression. **b**, **c** A low dosage of Avastin significantly blocked cellular-migration activity of hepatoma cells induced by *SPZ1-GFP* and *FlAG-TWIST1* coexpression, as underscored by wound-healing and Transwell migration assays performed in SK-Hep1 and Huh 7 cells. **d** A low dosage of Avastin significantly inhibits the proliferation and metastasis of xenografts of SK-Hep1 cells induced by coexpression of *SPZ1-GFP* and Flag-TWIST1 in vivo. Avastin reduced the tumorigenic activity 5–6 weeks after exposure to each drug. **e** Effect of Sorafenib and Avastin on the relative photon emission of SK-Hep1 cells in nude mice with coexpressed *SPZ1-GFP* and *Flag-TWIST1* (*n* = 5). **f** Statistical analysis of the survival curves of metastatic mice injected with SK-Hep1 cells transfected with *SPZ1-GFP* and *Flag-TWIST1* using the Kaplan–Meier method (*n* = 10). **g** Working hypothesis of the mechanism by which TIP60 regulates the putative complex of SPZ1-TWIST1 and induces metastasis of liver tumors. Cyto cytoplasm, Nuclei nuclear fraction, RTK receptor tyrosine kinase signaling, KAc lysine acetylation, PCNA proliferating cell nuclear antigen, Ki67 proliferation-related Ki-67 antigen

Considering the high proliferation and metastatic rates of hepatoma cells induced by the SPZ1–TWIST1 axis, we next evaluated the potential effects of inhibitors or modulators of upstream (RTK signaling) [11] or downstream (VEGF signaling involving the SPZ1–TWIST1 complex) pathways. Hepatoma cells were treated with an RTK inhibitor (Sorafenib) or a monoclonal anti-VEGF antibody (Avastin), both in vitro and in vivo, at doses employed in clinical trials. Sorafenib treatment slightly decreased SPZ1 and TWIST1 expression in Huh 7 and SK-Hep1 cells and their proliferation compared with vehicle-treated cells (Fig. 7a and Supplementary Figure S8a).

Avastin treatment significantly attenuated the expression of EMT markers, SPZ1, and TWIST1 in Huh 7 and SK-Hep1 cells and their proliferation compared with vehicle-treated cells. Co-treatment with sorafenib and bevacizumab markedly decreased the migration and invasion of hepatoma cells in vitro compared with vehicle treatment, as shown in the wound-healing (Fig. 7b, Supplementary Figure S8b, and S8c) and transwell (Fig. 7c and Supplementary Figure S8d) assays. To evaluate cell mobility and migration in vivo, SK-Hep1 cells with stable red fluorescent protein (RFP) expression as well as expression of both SPZ1 and TWIST1 were injected into nude mice. Mice injected with RFP-SK-Hep1-SPZ1-TWIST1 showed increased global proliferation and metastasis of tumor cells compared to mice injected with RFP-SK-Hep1 cells. Avastin treatment suppressed in vivo cell proliferation and metastasis within four weeks in mice injected with RFP-SK-Hep1-SPZ1-TWIST1 cells (Fig. 7d, e). Such mice showed shorter survival than those injected with RFP-SK-Hep1 cells. Avastin treatment improved the survival of nude mice injected with SK-Hep1 cells that expressed high levels of SPZ1 and TWIST1 (Fig. 7f).

## Discussion

‎In the present study, we identified a bHLH–ZIP transcriptional heterodimeric complex, SPZ1‒TWIST1, as an upstream regulator of EMT. SPZ1 functionally interacts with TWIST1 to promote metastatic activity and tumorigenesis. Further, acetylation of SPZ1 induced by TIP60 leads to the formation of the SPZ1–TWIST1 complex and its nuclear translocation, which consequently activates VEGF expression in hepatoma cells. Neutralization of VEGF expression abolishes the EMT activity induced by the SPZ1–TWIST1 complex. These results suggest that SPZ1 is an important regulator of tumor metastasis and indicate that VEGF, which is an important downstream regulator of SPZ1–TWIST1 signaling, may be a therapeutic target for SPZ1-induced tumors.

We have provided evidence of regulatory mechanisms involving the TWIST1-SPZ1 complex. First, we showed that the bHLH domain of SPZ1 functionally interacts with the WR domain of TWIST1, which is known to form a hetero- or homodimer complex to regulate early embryonic development as well as cell lineage determination and differentiation [9, 20, 21]. SPZ1 expression alone is known to activate the cell proliferation marker PCNA, leading to SPZ1 homodimer-regulated cell proliferation activity [11]. SPZ1 expression alone was shown to activate TWIST1 expression and initiate EMT [13], implying that SPZ1 may, to some extent, exert a regulatory effect on tumor progression induced by TWIST1 expression. In contrast to the original observation of proliferation regulation by its homodimer, SPZ1 activates and interacts with TWIST1 to form another functional bHLH dimer, which provides a plausible explanation for the mechanism by which SPZ1 transactivates TWIST1 expression during tumor progression. TWIST1 overexpression alone or co-expression with SPZ1∆B in Hep 3B cells showing low SPZ1 expression did not significantly activate EMT markers' expression, indicating the importance of the SPZ1–TWIST1 complex in tumor cell metastasis.

Second, we showed that the histone acetyltransferase TIP60 regulates SPZ1 and subsequent EMT. SPZ1 was shown to associate with and be acetylated by TIP60, which was required for SPZ1 recruitment to downstream regions of the VEGF promoters. Histone acetyltransferases (HATs) have been shown to be involved in cancer progression [22, 23]. The related HATs p300 and CBP have also been reported as key TWIST1 coactivators that promote EMT progression by repressing E-cadherin and p53 expression[17, 24]. TWIST1 was shown to be expressed in invasive primary tumors in *SPZ1* transgenic mice and in noninvasive neoplastic lesions, suggesting that TWIST1 exerts a positive regulatory effect on target genes involved in tumor initiation and progression [25, 26]. Acetylation of TWIST1 by TIP60 is reportedly essential for the recruitment of BRD4-mediated RNA-Pol II transcription machinery and expression of the downstream gene *WNT5A*, suggesting that TWIST1-BRD4 can positively promote the expression of downstream oncogenic genes [18]. In the present study, hepatoma cells treated with the TIP60 inhibitor TH1834 or transfected with the TWIST1 AC2 mutant showed loss of acetylation and nuclear localization of both TWIST1 and SPZ1. This demonstrated that acetylation by TIP60 is essential for the formation and nuclear localization of the SPZ1-TWIST1 complex. Our results suggest a mechanism regulating the SPZ1–TWIST1 complex through TIP60-mediated acetylation of critical lysine residues in both proteins (K73 and K76 in TWIST1; K369 and K374 in SPZ1). No experimental or structural modeling data are available to explain the regulation of interaction in the TIP60-SPZ1-TWIST1 axis including BRD4 recruitment. One possible mechanism is that TIP60 mediates acetylation of SPZ1, which interacts with TWIST1. TWIST1 might associate with the RNA polymerase II elongation complex. For example, Shi et al. [18]. reported that the NLS2 domain (aa 73–77) in TWIST1 interacted with the bromodomain of BRD4, which could associate with P-TEFb in the RNA-Pol II elongation complex and result in upregulation of *VEGF* gene expression. Interaction of TWIST1 with SPZ1 may also be mediated by the above NLS domain. The lysine residues in the NLS domain in TWIST1 may be critical for recruitment of SPZ1 to regulate activation of the *VEGF* gene promoter [12]. These speculations need to be experimentally verified in future studies.

Finally, in the present study, expression of the downstream oncogenic activator VEGF was upregulated by the SPZ1–TWIST1 complex through its interaction with BRD4 in tumor cells. Blocking or mutating the acetylation sites on SPZ1 or TWIST1 led to the disruption of the SPZ1–TWIST1 complex and attenuation of VEGF expression. Our study is the first to demonstrate that the TWIST1 complex positively regulates EMT progression via upregulation of its downstream target gene *VEGF*, which is an important tumor metastasis regulator and promoter of several physiological and pathological events including angiogenesis, vascular hyperpermeability, cancer metastasis, and cancer stem cell transition [27, 28]. It has been proposed that VEGF signaling regulates EMT and angiogenesis in the tumor microenvironment during tumor initiation and progression [1, 29]. Although several types of cells in the tumor microenvironment are believed to secrete VEGF including tumor cells, macrophages, and fibroblasts, the regulation of this event in secretory cells is unknown [1]. In the present study, tumor-initiating cells are the major source of secreted VEGF, which remodels the tumor microenvironment and confers more malignant attributes to the tumors. Blockade of secreted VEGF using a neutralizing antibody attenuates the metastatic behavior of tumor cells.

A model showing the mechanism by which SPZ1 exerts its effect on HCC is shown in Fig. 7g. In this model, SPZ1 signaling provides a link between RTK signaling [11] and HCC tumorigenesis, during which chronic oncogenic exposure to mitogens activates SPZ1 expression and the formation of homodimeric SPZ1, thus accelerating cell proliferation by deregulating cell-cycle control in the liver. Further, the SPZ1 homodimer activates TWIST1 expression and is acetylated by TIP60 forming the SPZ1–TWIST1 heterodimeric complex, which activates the expression of downstream EMT markers and initiates tumor metastasis.

In summary, we have shown that SPZ1 expression promotes angiogenesis, EMT, and metastasis by interacting with TWIST1 and activating it. Along with the oncosuppression induced by the formation of complexes of TWIST1 with other epigenetic regulators, the acetylated SPZ1–TWIST1 complex activates VEGF expression, thus inducing changes in tumor cells and the tumor microenvironment. These results highlight an important regulatory role of SPZ1 in promoting angiogenesis and invasion and indicate its potential as a therapeutic target in angiogenesis and metastasis.

## Materials And Methods

### Animals and cell culture

Male BALB/c nu/nu mice were obtained from the National Laboratory of Animal Breeding and Research Center (Taipei, Taiwan) and housed according to the protocols of the Animal Center of the Kaohsiung Medical University (Kaohsiung, Taiwan). *Spz1* transgenic mice were generated by Dr. Hung Li (Academia Sinica, Taipei, Taiwan) [11, 12]. Human hepatoma cell lines (Hep G2, Hep 3B, SK-Hep1, Huh 7, PLC/PRF/5, and HA 22T) were obtained from the American Type Culture Collection (ATCC; Manassas, VA, USA) and maintained according to ATCC protocols. The study was conducted with approval (IACUC-104181) from the ethics committee of Kaohsiung Medical University.

### Plasmids, cell Lines, and other materials

The full-length *SPZ1* cDNA was amplified from a human testis cDNA library (GIBCO/BRL, Cheshire, UK) using PCR. The *SPZ1* cDNA and a mutant *SPZ1* were each subcloned into the *pEGFP/C1* vector (or *pEYFP/C1*) (Clontech) to express a GFP-tagged SPZ1 (or YFP-tagged SPZ1). Full-length TWIST1 (a gift from Dr. Yang MH, NYMU, Taipei, Taiwan) [30] and mutants were inserted into the HA (or CFP and mCherry)-tagged TWIST1 [31]. The *PLKO.1.puro* or*.neo* vector was used as a backbone for shRNAi constructs targeting *SPZ1* (target sequence, ACTTGTCAGTCATGATCAATC) [19] or *TWIST1* (target sequence, 1. AGCTGAGCAAGATTCAGACCC; 2. GCATTCTGATAGAAGTCTGAA; 3. CCTGAGCAACAGCGAGGAAGA). The SK-Hep1, Hep G2, Hep 3B, HA 22T, and PLC cell lines were subcultured and maintained according to ATCC protocols. Transfection was performed using a Lipofectamine transfection kit (GIBCO/BRL).

### Confocal fluorescence resonance energy transfer (FRET) analysis

The *SPZ1* cDNA was subcloned into the *pEYFP/C1* vector (Clontech) to express YFP-tagged SPZ1. Full-length TWIST1 cDNA was inserted into a *pECFP/C1* vector to express CFP-tagged TWIST1. The SK-Hep1 and Huh-7 cells were seeded on coverslips. One day later, the two constructs were cotransfected into cells. The experiments were performed using an Olympus FV1000 confocal microscope. When TWIST1–CFP is excited by 422 nm (cyan) and the two molecules are within 10 nm of each other, energy can be transferred from the excited CFP to the SPZ1-YFP (485 nm, green), leading to emission of yellow light by the YFP (530 nm, white), which is then detected independently of the blue light that excited CFP. The FRET efficiency (*E*) is the quantum yield of the energy transfer transition, i.e., the fraction of energy transfer event occurring per donor excitation event as follows:

$${{E = k}}_{{\mathrm{ET}}}{{/K}}_{\mathrm{f}}{{ \,+\, K}}_{{\mathrm{ET}}}{{ \,+\, \Sigma }}^{{{K_i}}}{{,}}$$

where *k*_ET_ is the rate of energy transfer, *k*_f_ is the radiative decay rate, and *k*_i_ is the rate constant of any other de-excitation pathways [32].

### ELISA to measure human VEGF concentrations in culture medium

VEGF concentration was measured in the culture medium using a human VEGF ELISA Kit (Invitrogen), according to the manufacturer’s instructions. Human hepatoma cells overexpressing *SPZ1* (or *TWIST1*) or transfected with shRNA were seeded in 6-well culture plates and incubated at 37 ℃. Twenty-four hours later, the conditioned medium was collected and centrifuged at 3000 r.p.m. for 20 min to remove cell debris. The supernatants were then subjected to ELISA in triplicate. VEGF concentrations were computed with reference to the standard curve derived from the purified VEGF supplied in the ELISA Kit.

### Wound-healing assay

The human hepatoma cells overexpressing *SPZ1* (or *TWIST1*) or transfected with shRNA that were seeded in 10-cm culture plates were scratched using a pipette tip to create a gap, followed by incubation at 37 °C and imaged every 12 h using a digital camera attached to a microscope. For each gap, the average width was computed from three measurements taken at the top, middle, and bottom end of the microscopic field.

### Transwell invasion assay

The Transwell invasion assay was performed using a Transwell chamber (Life Technologies) with a Matrigel-coated filter. Human hepatoma cells (1 × 10^5^) overexpressing *SPZ1* (or *TWIST1*) or transfected with shRNAi were added to 250 μL of serum-free media and plated onto the upper chamber of the Transwell. The upper chamber was then transferred to a well containing 500 μL of media supplemented with 10% FBS and incubated for 18 h. Cells may actively migrate from the upper to the lower side of the filter using FCS as an attractant. Cells on the upside were removed using cotton swabs, and the invasive cells on the lower side were fixed, stained with a 0.2% crystal violet solution, and counted under a light microscope. The experiment was repeated three times.

### Determination of tumor growth and metastasis by IVIS analysis

To perform a tumor growth and metastasis analysis in vivo, vector- or SPZ1 mutant-transfected SK-Hep1-RFP cells (1 × 10^6^) were injected into liver tissues of 6–8-week-old male nude mice, and the images were monitored using IVIS each week.

For the inhibition of VEGF using a monoclonal antibody, RFP-expressing SK-Hep1 cells (1 × 10^7^) transfected with vector or *SPZ1* (or *TWIST1*) mutants or specific shRNAi alone, as well as both *SPZ1*–*TWIST1* shRNAi, were injected into tail veins of 6‒8-week-old male nude mice. Two weeks later, mice that were injected with SPZ1-transfected SK-Hep1-RFP cells were divided into four groups; one group was designated as the control and the other groups were treated separately with Sorafenib (20 mg/kg/day, orally), Avastin (5 mg/kg/day, IV), and both Sorafenib and Avastin. Mice were imaged every week in the prone position in an IVIS200 Imaging System (Caliper Life Sciences, Hopkinton, MA, USA). Data were acquired and analyzed using the Living Image software, version 4.2.

### Statistical analysis

Quantitative variables are presented as the mean ± s.d. The significance of differences was determined using a two-sample *t*-test. Differences with *P* value < 0.05 were considered significant. (Additional materials and methods are described in the Supplementary Information section.)

Patients, Western blotting, and immunohistochemical analysis, luciferase reporter assay, ChIP assay, Two-step ChIP assay, EMSA, real time PCR, and anchorage-independent growth assay were described in the Supplementary Information section.

## Electronic supplementary material

SUPPLEMENTAL INFORMATION

Supplemental Figure 1

Supplemental Figure 2

Supplemental Figure 3

Supplemental Figure 4

Supplemental Figure 5

Supplemental Figure 6

Supplemental Figure 7

Supplemental Figure 8
