# Supplementary material for: TIP60-dependent acetylation of the SPZ1-TWIST complex promotes epithelial–mesenchymal transition and metastasis in liver cancer
Source: Oncogene. 2018 Aug 28;38(4):518–32. doi: 10.1038/s41388-018-0457-z (PMC6345675; doi:10.1038/s41388-018-0457-z)
Supplement: Supplementary file 1 — SUPPLEMENTAL INFORMATION [file 41388_2018_457_MOESM1_ESM.docx]

**Supplemental information**

**Materials and Methods**

**Patients**

This study enrolled 220 patients with hepatocellular carcinoma (HCC) from July 2007 to July 2014 from two medical centers in Taiwan; Chung Ho Memorial Hospital (186 patients) and Changhua Christian Hospital (34 patients), with hepatitis B virus (HBV) and/or hepatitis C virus (HCV) infection, and 153 patients without HCC, of whom 85 were infected with HBV or HCV and 68 were not infected with either virus. The study of human subjects was approved by the Institutional Review Board of Kaohsiung Medical University (KMUHIRB-20130052; Kaohsiung, Taiwan).

**Western Blotting and Immunohistochemical Analysis**

Western blotting and immunohistochemical (fluorescence) staining were performed as described previously.^1,2^ The primary antibodies used in this study were Cyclin D1, E-cadherin, E2F1, and Vimentin, and actin polyclonal antibodies (Merck, Darmstadt, Germany), HA and GFP monoclonal antibodies (Merck, Darmstadt, Germany), FITC-conjugated anti-rabbit IgG, rhodamine-conjugated anti-mouse IgG, and alkaline phosphatase-conjugated anti-rabbit IgG antibody (1:500 dilution; Jackson ImmunoResearch Laboratories, West Grove, PA, USA), Ki67 goat polyclonal antibody (1:200 dilution; Santa Cruz Biotechnology, Dallas, TX, USA), SPZ1, VEGF, TWIST1 rabbit polyclonal antibody (1:200 dilution; Santa Cruz Biotechnology). BRD4 and TIP60 rabbit polyclonal antibody (1:200 dilution; Novus), acetylated-Lysine rabbit polyclonal antibody (1:500 dilution; Cell Signaling; Beverly, MA, USA). The Fibronectin and N-cadherin (1:500 dilution) primary antibody were obtained from GeneTex International Corp. All experiments were repeated at least three times.

**Luciferase Reporter Assays**

The VEGF promoter (between positions −991 and +20) was cloned from human placenta genomic DNA and was used to construct a pGL3 luciferase reporter plasmid.^3^ The expression constructs and two reporter constructs, pSV40-Rluc and pGL3-VEGF/Fire luciferase (Promega Co., Madison, WI, USA), were cotransfected with SPZ1 and TWIST1 into 2 × 10^5^ Hep 3B cells. The cells were harvested 16 h after the transfection, and the relative luciferase activity was measured according to the manufacturer’s instructions. All data are expressed as the mean ± s. d. of at least three experiments.

**Chromatin Immunoprecipitation (ChIP) Assays**

The chromatin immunoprecipitation (ChIP) assays were performed as described previously. All data are expressed as the mean ± s. d. of at least three experiments. The VEGF promoter fragment was amplified with the following primers: Primer 1, 5′-TTC ACT GGG CGT CCG CAG AG-3′; Primer 2, 5′-TGC CCC AAG CCT CCG CGA TC-3′.

**Two-step Chromatin Immunoprecipitation (ChIP) Assays**

Two-step ChIP assays were performed as described[2]. Briefly, SK-Hep1 cells were treated with 1.0% formaldehyde to crosslink proteins to DNA for 10 min at room temperature. Cells were spin down and lysed with RIPA lysis buffer. Genomic DNA was sheared by sonication for 10 min at the M2 intensity level to acquire optimal DNA fragment size of ~300 bp. Immunoprecipitation was achieved by the addition of anti-GFP (SPZ1) antibody or Normal Rabbit IgG to the samples with rotation at 4 °C overnight. Protein A/G agarose beads (25 ul) were added to the samples and incubated for 1 h at 4 °C with rotation. Beads were washed with 10 mM PBS and then eluted with 0.1M glycine solution (pH 2-3). Eluted proteins from first immunoprecipitation step ere then precipitated by addition of anti-FLAG (TWIST1) antibody to samples or Normal Rabbit IgG as negative control sample and followed by second-round IP. Next day, samples were washed by 10 mM PBS and then eluted with TE buffer for quantitative PCR. The VEGF promoter fragment was amplified with the following primers: Primer 1, 5′-TTC ACT GGG CGT CCG CAG AG-3′; Primer 2, 5′-TGC CCC AAG CCT CCG CGA TC-3′.

**Electrophoretic mobility shift assay (EMSA)**

The DNA-binding reaction was performed as previously described [2], with minor modifications: the SPZ1-GFP and mutant proteins (or the TWIST1-mcherry and mutant proteins) were purified using a column packed with SulfoLink Coupling Gel, and the [^32^P]-labeled oligonucleotides were incubated in buffer containing 10 mM Tris-HCl, 1 mM EDTA, 100 mM NaCl, 2 mM dithiothreitol (DTT) and 10% glycerol. A double-stranded oligonucleotide (G-rich sequence) containing the VEGF promoter elements (−96 to −70 bp) was used as a probe to interact with the purified recombinant proteins. The antibody against GFP or mCherry was added 30 min before mixing the purified proteins with the oligonucleotides for a supershift assay. Further, 200 ng of purified protein was incubated with 5,000 cpm ^[32^P]-labeled oligonucleotides, 2 μg poly (dI:dC), and BSA (1μg/mL) for 30 min at room temperature. The DNA/protein mixtures were separated on a 5% polyacrylamide gel (30:1 bis-acrylamide in 0.5× TBE) as described [2].

**Real-Time PCR**

The expression of *SPZ1* and *TWIST1* mRNA in hepatoma cells and cells from cancer patients was quantified using an SYBR Green Quantitative RT-PCR kit (Invitrogen) as described previously[2]. Total RNA was extracted from tumor mass using the TRIzol reagent (Invitrogen) and then transcribed into single-stranded cDNA (Invitrogen) for PCR amplification on a 7900HT Thermocycler (Thermo Fisher Scientific, Waltham, MA, USA). All procedures and data analysis were performed according to the manufacturers’ instructions. The cells were transfected with an empty pEGFP vector and samples from healthy subjects and drug-treated patients were analyzed for comparison. All data are expressed as the mean ± s. d. of at least three experiments.

**Anchorage Independent Growth Assays**

Cells (10^4^ or 5 × 10^3^) in 1 mL of a culture medium were mixed with an equal volume of 0.6% top agar and plated onto 60-mm culture dishes with 0.5% bottom agar. The plates were incubated at 37°C for 2 weeks. Colonies were visualized by staining with 0.05% crystal violet acetate, and only those larger than 0.5 mm were counted. The culture medium was replaced every 3 days. All data are expressed as the mean ± s. d. of at least three experiments.

**References:**

1. Chiou, S. S., Wang, L. T., Huang, S. B., Chai, C. Y., Wang, S. N., Liao, Y. M., Lin, P. C., Liu, K. Y., and Hsu, S. H. (2014). Wntless (GPR177) expression correlates with poor prognosis in B-cell precursor acute lymphoblastic leukemia via Wnt signaling. Carcinogenesis. 35 (10) p.2357–2364.

2. Hsu, S. H., Hsieh-Li, H. M., Huang, H. Y., Huang, P. H., and Li, H. (2005). bHLH-zip transcription factor Spz1 mediates mitogen-activated protein kinase cell proliferation, transformation, and tumorigenesis. Cancer research *65*, 4041-4050.

3. Hsu, S. H., Hsieh-Li, H. M., and Li, H. (2004). Dysfunctional spermatogenesis in transgenic mice overexpressing bHLH-Zip transcription factor, Spz1. Experimental cell research *294*, 185-198.

**Supplemental Figures**

**Figure S1.** Protein profiles of immunoprecipitates with anti-mCherry antibody from lysates of TWIST1-mCherry or mCherry transfected SK-Hep1 cells. (a) The anti-TWIST1 immunoprecipitants were analyzed by two-dimensional electrophoresis as described in Materials and Methods. (b) Oligopeptide (SQKDISETLGNNGVGFQTQPNNEVSAK) of SPZ1 (number 358 in panel A) generated by digestion with TPCK-trypsin was sequenced by LC Mass/Mass in anti-TWIST1 immunoprecipitates as described in Materials and Methods.

**Figure S2.** Interaction of SPZ1 and TWIST1. (a) SPZ1-YFP colocalized with TWIST1-CFP analyzed by fluorescence resonance energy transfer (FRET) in Heh 7cells. Green, SPZ1-YFP; Cyan, TWIST1-CFP; white, FRET signals (Lower panels). The oblique line marks the analyzed sites for FRET signaling. Nuc and Cytosol indicate nuclear fraction and cytoplasmic fraction, respectively. (b) SPZ1 interactes with TWIST1 weakly in normal mouse livers (left panel) and human patient- derived normal livers (right panel).

**Figure S3.** Identification of domains required for interaction between SPZ1 and TWIST1 by coimmunoprecipitation-Western blots (CoIP–WB) analysis. IP, immunoprecipitation; WB, western blot. (a) TWIST1 does not interact with SPZ1ΔB mutant in Huh 7 cells. V, vector; FL, full-length SPZ1; ΔB, SPZ1 without bHLH domain; ΔL, SPZ1 without leucine zipper. *HA-TWIST1* and *GFP-SPZ1* variants were transfected into Huh 7 cells and assayed by CoIP –WB. (b) SPZ1 does not interact with ΔWR TWIST1 mutant in Huh 7 cells. V, vector only; FL, full-length SPZ1; ΔWR, TWIST1 without WR domain; ΔB, TWIST1 without bHLH domain. *SPZ1-GFP* and *TWIST1-mCherry* mutants were co-transfected into Huh 7 cells and assayed by CoIP–WB. (c) and (d) Coexpression of various *SPZ1-GFP* mutants and *TWIST1* does not activate the proliferation-related protein levels (cyclin D1, E2F1, PI3K, and AKT), EMT markers (VEGF, E-cadherin, Snail1, and Slug) and mesenchymal markers (N-cadherin, Fibronectin, and Vimentin) in Hep 3B cells. SPZ1ΔB shows no expression of theses marker genes. (e) Coexpression of *SPZ-1-GFP* and various *TWIST1* mutants, but not individually in SK-Hep1 cells, activates expression of EMT markers (VEGF, E-cadherin, Snail1, and Slug) and mesenchymal markers (N-cadherin, Fibronectin, and Vimentin).

**Figure S4.** Colocalization of SPZ1 with VEGF or CD31. (a) SPZ1 colocalizes with VEGF in tumor regions induced by SK-Hep1 cells transfected with GFP- SPZ1 or SPZ1-specific shRNAi. Green, SPZ1; red, VEGF; yellow, merge. (b) Both Spz1 and VEGF showed a significant colocalization in livers from *Spz1* transgenic mice. Green, Spz1; Red, VEGF; HE, hematoxylin and eosin stain. T, tumor region; N, normal region. The square box indicates the area analyzed. (c) Expression of SPZ1 and CD31 in tumor tissues derived from SK-Hep1 cells transfected with GFP-tagged SPZ1 or SPZ1-specific shRNAi. Green, SPZ1; red, CD31; yellow, merge. (d) Both Spz1 and CD31 showed a significant colocalization in liver from *Spz1* transgenic mice. Green, Spz1; red, CD31; HE, hematoxylin and eosin stain. The square box indicates the area analyzed.

**Figure S5.** Effect of TIP60 inhibitor. (a) and (b) TIP60 acetylation inhibitor (TH1834; 20μM) decreased the expression of SPZ1 and blocked the interaction between SPZ1 and TWIST1 by inhibiting TIP60-mediated acetylation in SK-Hep1 and HA 22T cells. (c) and (d) Effect of TH1834 on expression of proliferation-related proteins (c) and EMT-related proteins (d). SK-Hep1 and HA 22T cells treated with TIP60 inhibitor. (e) Huh 7 cells treated with TH1834 showed luciferase activity of *VEGF* promoter and the recruitment of SPZ1 on *VEGF* promoter. a, *P*<0.001. (f) SK-Hep1 and HA22T cells treated with TIP60 inhibitor decreased significantly in wound-healing migration activity. The quantitation is shown in Figure 4d.

**Figure S6.** Characterization of domain structures of SPZ1 and TWIST1 for interaction. (a) Interaction of FLAG-tagged TWIST WT and its mutants and SPZ1-GFP was evaluated by immunoprecipitation-Western blot (IP-WB). The acetylation mutation site of TWIST1 (AC2) significantly decreased the expression of EMT markers in SK-Hep1 cells. (b) *FLAG-TWIST1 AC2* lost the interaction with *SPZ1-GFP* in Huh 7 cells. (c) *FLAG-TWIST1 AC2* reduced the interaction with *SPZ1-GFP* in the cytoplasm and nuclei in Huh 7 cells. Green, TWIST; Red, SPZ1; Blue, DAPI. (d) and (e) Forced expression of *FLAG-TWIST1 AC2* and *SPZ1-GFP* abolished the recruitment of SPZ1-GFP to the *VEGF* promoter in SK-Hep1 cells (d) and Huh 7 cells (e). (f) Forced *TWIST1 AC2* expression significantly decreased the release of VEGF into the medium in SK-Hep1 and Huh 7 cells. The results are shown as the mean ± s. d. a and b, *P* < 0.001. Each experiment was repeated at least three times.

**Figure S7.** SPZ1-GFP AC2 decreases the expression of EMT-related protein markers and migration and invasion in SK-Hep1 and Huh7 cells. (a) The acetylation site mutation of SPZ1 (SPZ1-GFP AC2) abolished the expression of EMT-related marker proteins in SK-Hep1 and Huh7 cells. (b) The acetylation site mutation of SPZ1 (SPZ1-GFP AC2) repressed cell migrationas determined by wound-healing assay described in Materials & Methods. (c) The acetylation site mutation of SPZ1 (SPZ1-GFP AC2) repressed the cell invasion activity determined by trans-well invasion assay. The results are shown as the means ± s. d. *a*, *P*< 0.001. Each experiment was repeated at least three times.

**Figure S8.** Effect of antibodies against VEGF and inhibitors of RTK and VEGF on proliferation, migration and invasion activities. (a) SK-Hep1 cells treated with low dosage of RTK and/or VEGF inhibitor reduced the expression of proliferation-related proteins and EMT-related proteins. (b) SK-Hep1 cells treated with low dosage of RTK or/and VEGF inhibitor decreased the wound-healing activity. Addition of DOX induced the expression of SPZ1. (c) Huh 7 treated with low dosage of RTK and/or VEGF inhibitor decreased the wound-healing activity. Addition of DOX induced the expression of SPZ1. (d) Huh 7 treated with low dosage of inhibitor of RTK and/or VEGF decreased the invasion activities. The results are shown as means ± s. d. *a*, *P*< 0.001. Each experiment was repeated at least three times.
