# Supplementary figures and images for: TIP60-dependent acetylation of the SPZ1-TWIST complex promotes epithelial–mesenchymal transition and metastasis in liver cancer
Source: Oncogene. 2018 Aug 28;38(4):518–32. doi: 10.1038/s41388-018-0457-z (PMC6345675; doi:10.1038/s41388-018-0457-z)

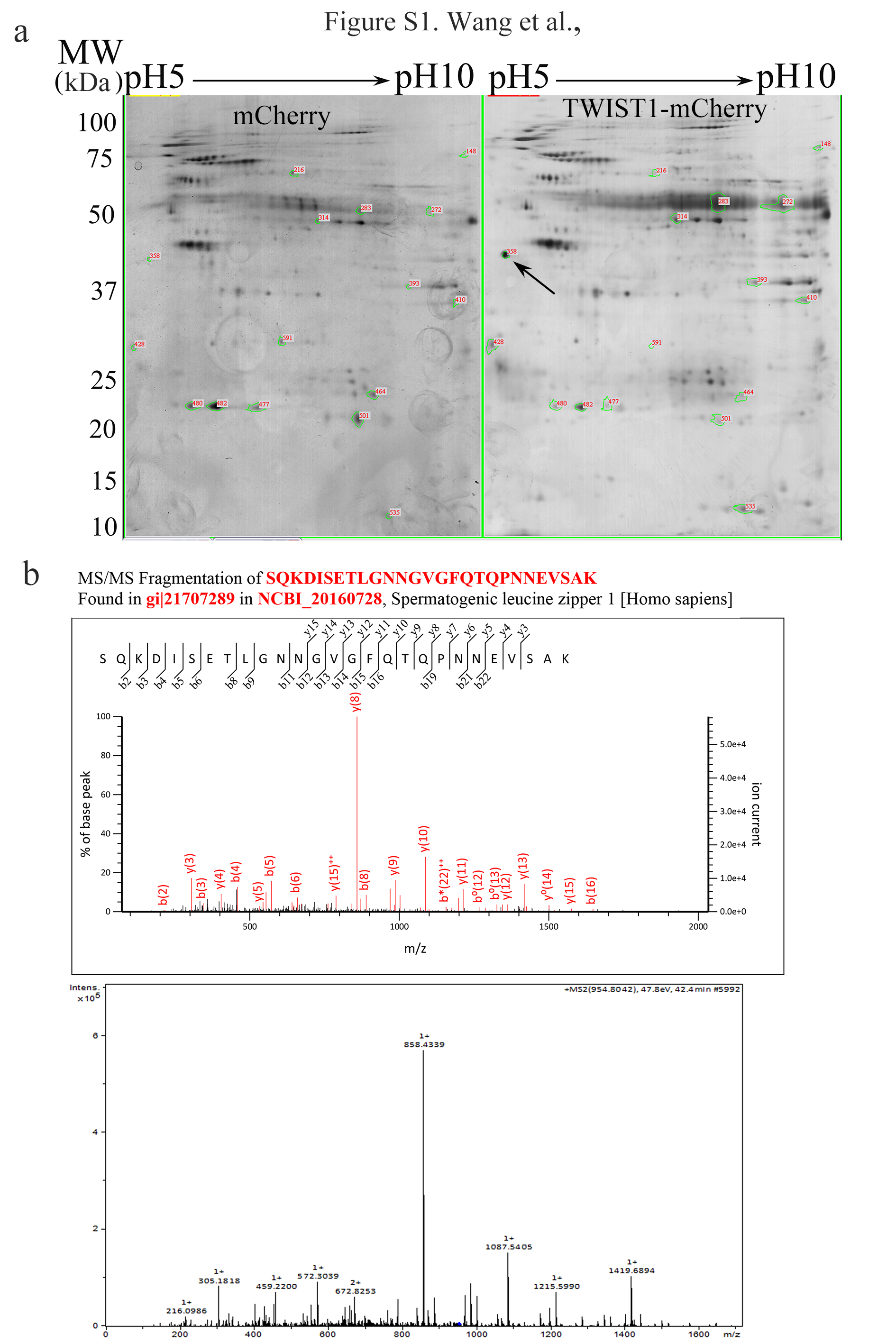

Supplement: Supplementary file 2 — Supplemental Figure 1 [file 41388_2018_457_MOESM2_ESM.tif]

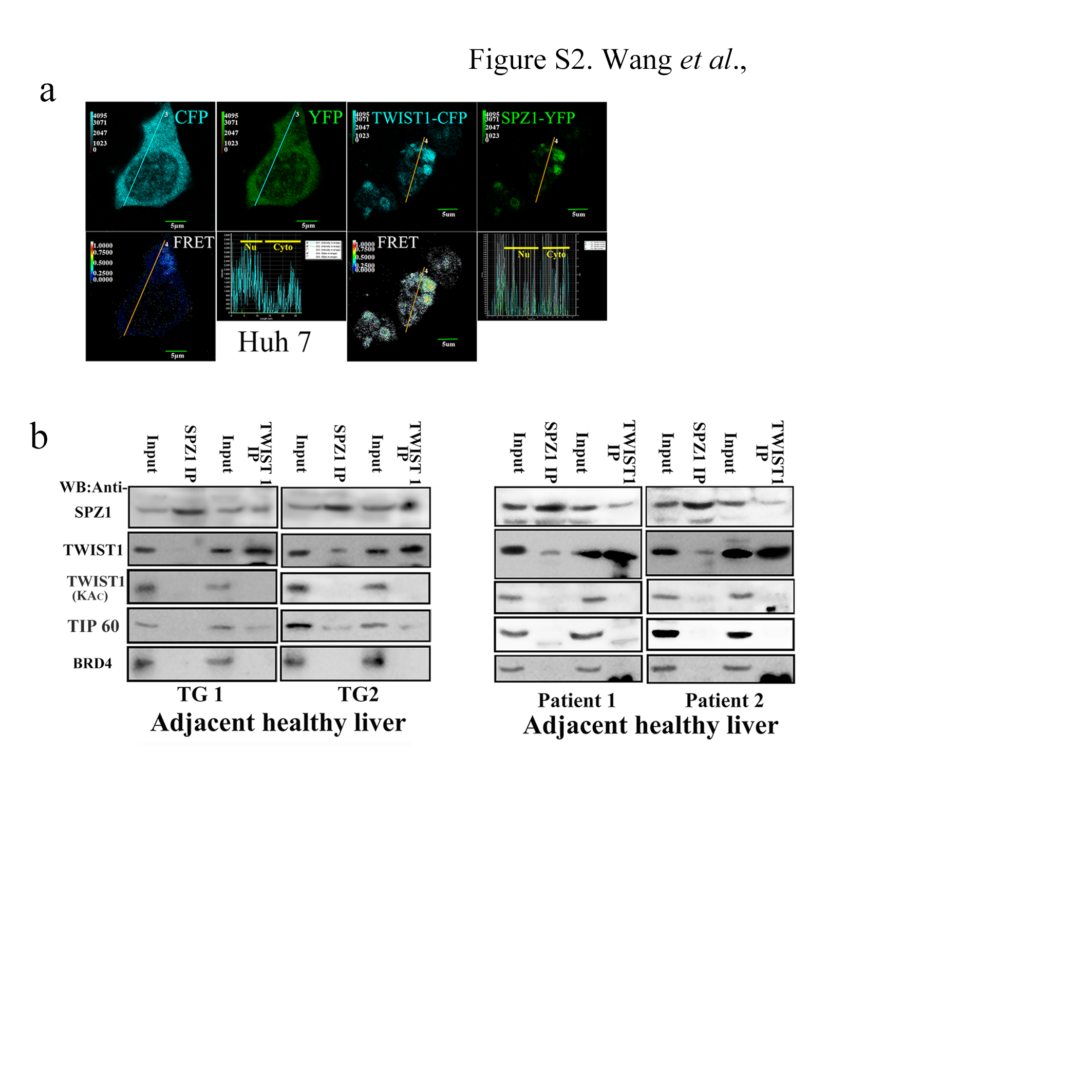

Supplement: Supplementary file 3 — Supplemental Figure 2 [file 41388_2018_457_MOESM3_ESM.tif]

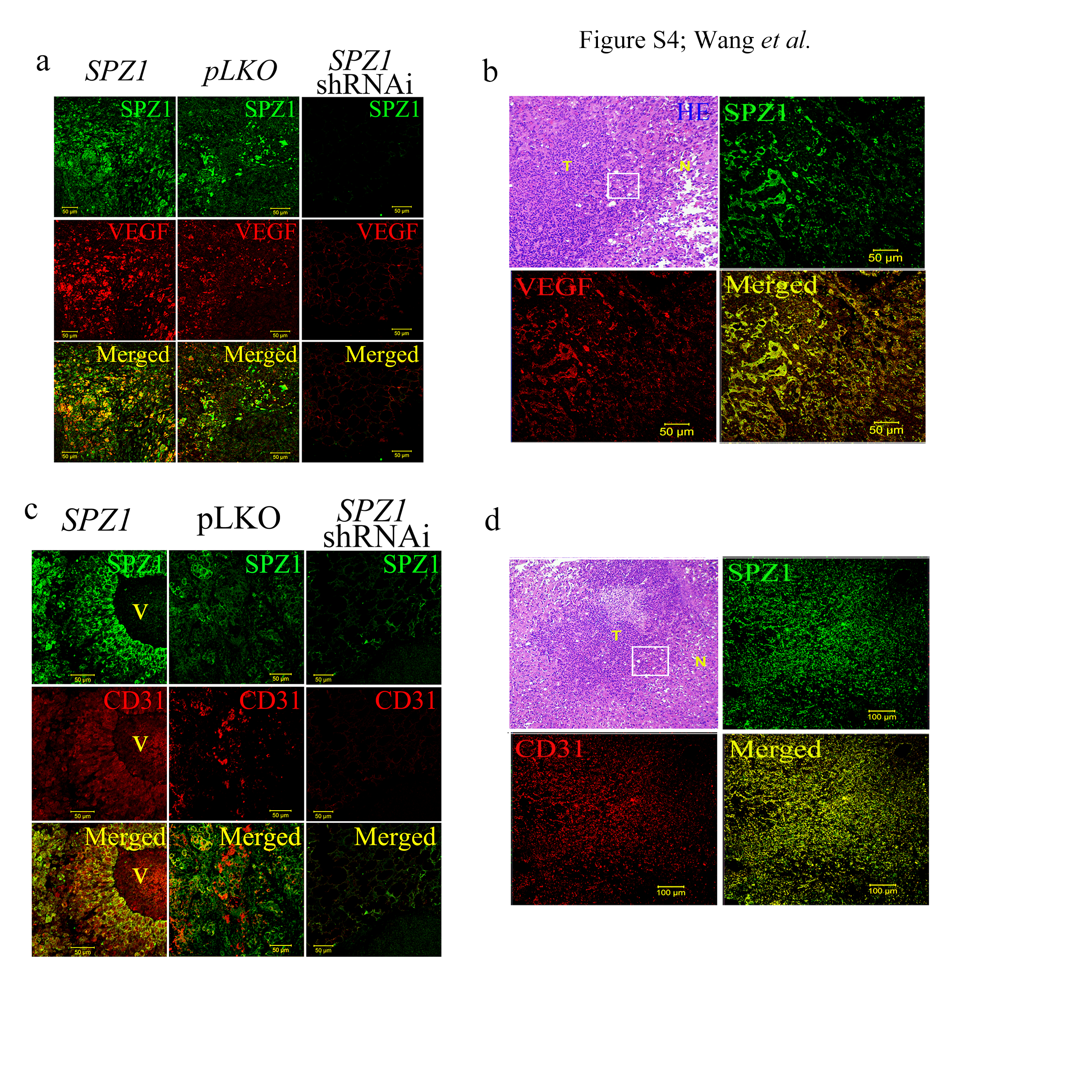

Supplement: Supplementary file 5 — Supplemental Figure 4 [file 41388_2018_457_MOESM5_ESM.tif]

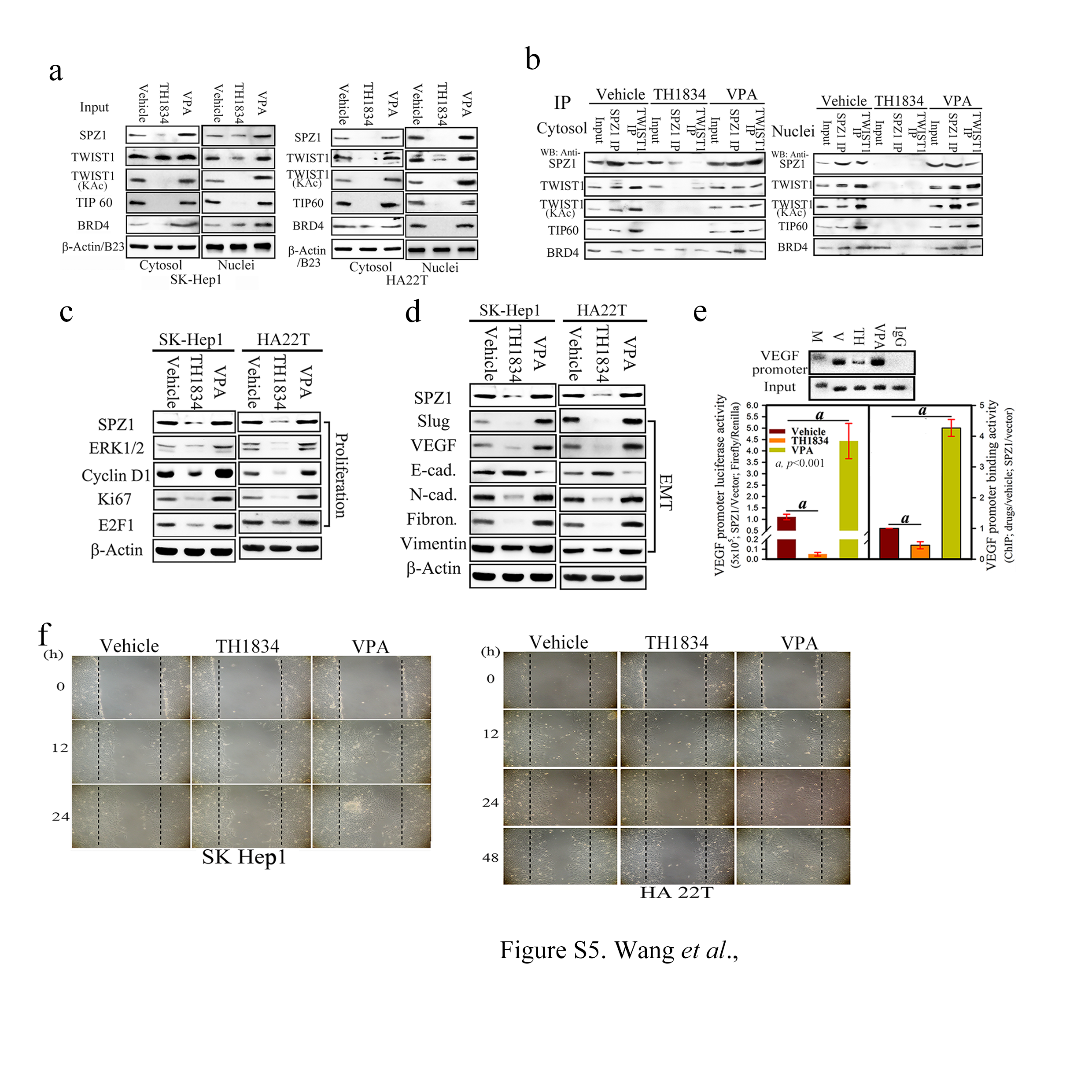

Supplement: Supplementary file 6 — Supplemental Figure 5 [file 41388_2018_457_MOESM6_ESM.tif]
